# Effect of Guanidinoacetic Acid Supplementation on Growth Performance, Rumen Fermentation, Blood Indices, Nutrient Digestion, and Nitrogen Metabolism in Angus Steers

**DOI:** 10.3390/ani14030401

**Published:** 2024-01-26

**Authors:** Simeng Yi, Sanlong Hu, Jinze Wang, Abudusaimijiang Abudukelimu, Yao Wang, Xiang Li, Hao Wu, Qingxiang Meng, Zhenming Zhou

**Affiliations:** State Key Laboratory of Animal Nutrition and Feeding, College of Animal Science and Technology, China Agricultural University, Beijing 100193, China; yisimeng97@163.com (S.Y.);

**Keywords:** guanidinoacetic acid, Angus steers, growth performance, digestive metabolism, nitrogen metabolism

## Abstract

**Simple Summary:**

This study investigated the effects of guanidinoacetic acid (GAA) supplementation on the growth performance, rumen fermentation, blood indices, nutrient digestion, and nitrogen metabolism of Angus steers. In a 130-day feeding experiment, steers receiving GAA at a conventional dose (0.8 g/kg) and a high dose (1.6 g/kg) exhibited significantly higher average daily weight gain and improved feed conversion efficiency compared to the control group without GAA. GAA supplementation also influenced rumen fermentation, with lower acetate levels, higher propionate levels, and higher acetate: propionate ratio in GAA groups. Blood analyses revealed elevated concentrations of urea, blood ammonia, GAA, creatine, and catalase, indicating improved creatine metabolism and antioxidant activity. A subsequent 3-day digestive metabolism experiment confirmed that the GAA-supplemented groups had increased dry matter and crude protein digestibility, as well as increased N retention and improved organismal nitrogen metabolism. In summary, dietary GAA supplementation at a 0.8 g/kg and 1.6 g/kg DM basis positively influenced growth performance, rumen fermentation, blood indices, nutrient digestion, and nitrogen metabolism in Angus steers, emphasizing its potential as a nutritional strategy.

**Abstract:**

Guanidinoacetic acid (GAA) functions as a precursor for creatine synthesis in the animal body, and maintaining ample creatine reserves is essential for fostering rapid growth. This study aimed to explore the impact of GAA supplementation on growth performance, rumen fermentation, blood indices, nutrient digestion, and nitrogen metabolism in Angus steers through two experiments: a feeding experiment (Experiment 1) and a digestive metabolism experiment (Experiment 2). In Experiment 1, thirty-six Angus steers (485.64 ± 39.41 kg of BW) at 16 months of age were randomly assigned to three groups: control (CON), a conventional dose of GAA (CGAA, 0.8 g/kg), and a high dose of GAA (HGAA, 1.6 g/kg), each with twelve steers. The adaptation period lasted 14 days, and the test period was 130 days. Weighing occurred before morning feeding on days 0, 65, and 130, with rumen fluid and blood collected before morning feeding on day 130. Experiment 2 involved fifteen 18-month-old Angus steers (575.60 ± 7.78 kg of BW) randomly assigned to the same three groups as in Experiment 1, with a 7-day adaptation period and a 3-day test period. Fecal and urine samples were collected from all steers during this period. Results showed a significantly higher average daily gain (ADG) in the CGAA and HGAA groups compared to the CON group (*p* = 0.043). Additionally, the feed conversion efficiency (FCE) was significantly higher in the CGAA and HGAA groups than in the CON group (*p* = 0.018). The concentrations of acetate and the acetate:propionate ratio were significantly lower in the CGAA and HGAA groups, while propionate concentration was significantly higher (*p* < 0.01). Serum concentration of urea (UREA), blood ammonia (BA), GAA, creatine, and catalase (CAT) in the CGAA and HGAA groups were significantly higher than in the CON group, whereas malondialdehyde (MDA) concentrations were significantly lower (*p* < 0.05). Digestibility of dry matter (DM) and crude protein (CP) and the nitrogen retention ratio were significantly higher in the CGAA and HGAA groups than in the CON group (*p* < 0.05). In conclusion, dietary addition of both 0.8 g/kg and 1.6 g/kg of GAA increased growth performance, regulated rumen fermentation and blood indices, and improved digestibility and nitrogen metabolism in Angus steers. However, higher doses of GAA did not demonstrate a linear stacking effect.

## 1. Introduction

Beef cattle contribute significantly to the meat industry, and enhancing the performance of beef cattle is essential for the development of the beef cattle breeding sector [1]. Creatine plays a crucial role in maintaining energy homeostasis in vertebrate muscle tissues [2] and is actively involved in muscle energy metabolism and protein synthesis [3,4]. Adequate creatine reserves are a prerequisite for rapid growth in animals [5]. But, creatine is found only in products of animal origin [6], and since cattle usually have a plant-based diet, they must synthesize creatine themselves [7]. Guanidinoacetic acid (GAA) is synthesized in the kidney from arginine and glycine and is the only precursor substance for creatine synthesis in animals [8]. Since, as an exogenous additive, GAA is more stable and cheaper than creatine [9], supplementation with GAA in livestock has been evaluated as an alternative to creatine [10,11,12,13]. Therefore, it is necessary to explore the efficacy of exogenously added GAA in ruminants.

Recently, GAA has been extensively studied as a supplement for ruminants; for example, the addition of 0.6 or 0.9 g/kg DM GAA improved growth performance and nutrient digestibility in bulls [14], and the addition of 1 g/kg DM GAA has the potential to improve growth performance and meat quality in lambs [15]. Moreover, the addition of GAA to the diet is able to produce more creatine while reducing arginine consumption in beef cattle [16]. This process involves not only energy metabolism but also N deposition (nitrogen metabolism) in the beef cattle organism [17]. In addition, GAA has been reported to enhance the antioxidant capacity of the rumen in ruminants [18]. In summary, GAA has the potential to improve growth performance in ruminants, but little is known about the effects of GAA on production performance and nitrogen metabolism in Angus steers; furthermore, it remains to be explored whether the addition of high doses of GAA would have a linear superimposed effect on the metrics in Angus steers.

Therefore, the present study utilized feeding experiments to investigate the effects of GAA on the growth performance, rumen fermentation, and blood-related indexes of Angus steers, and to investigate whether the addition of high doses of GAA had a linear superposition effect on the indexes of steers; it also utilized digestive metabolism experiments to investigate the effects of the addition of GAA on the digestibility of nutrients and the metabolism of nitrogen in Angus steers. The hypotheses of this study are the following. (1) Dietary supplementation with a conventional dose of GAA can improve the growth performance of Angus steers, while the addition of high doses of GAA has a linear superimposed effect on the growth performance of Angus steers. (2) Dietary supplementation with GAA positively affects the related indexes (creatine metabolism and antioxidant indexes) in the blood of Angus steers. (3) Dietary supplementation with GAA can significantly improve nutrient digestibility and body nitrogen metabolism.

## 2. Materials and Methods

This animal study was approved by the Experimental Animal Welfare and Animal Experimentation Ethics Committee of China Agricultural University. This study was conducted in accordance with the local legislation and institutional requirements.

### 2.1. Guanidinoacetic Acid Products

The guanidinoacetic acid used in this experiment is a white powdery product with an effective content of not less than 980 g/kg. The GAA products were donated by Beijing Junde Tongchuang Biotechnology Co., Ltd. (Beijing, China). 

### 2.2. Experimental Design and Animal Feeding Management

Experiment 1. Thirty-six 16-month-old Angus steers (485.64 ± 39.41 kg of BW) were selected and randomly assigned to 3 groups according to a one-factor randomized block design, with 12 steers per group. The groups were the control group (CON), the conventional dose guanidinoacetic acid group (CGAA), and the high dose guanidinoacetic acid group (HGAA). The addition levels of GAA (Beijing Junde Tongchuang Biotechnology Co., Ltd., Beijing, China) were 0 g/kg, 0.8 g/kg, and 1.6 g/kg on a DM basis, respectively. The feeding method of GAA was to premix it with concentrate and then mix it with the total mixed ration (TMR) for feeding. The common dose of GAA was determined based on previous studies [14,15,19], and the high dose was twice the common dose. The purpose was to determine whether the doubling of the addition level of GAA could bring a linear superposition of growth performance. The experimental diet used in this study was formulated according to the beef cattle nutritional requirements of NASEM (2016) [20]. The concentrate-to-roughage ratio of the diet was 40:60. The composition and nutritional level of the diet are shown in Table 1. This experiment was conducted in Beijing from January to June, with a pre-test period of 14 days and a formal test period of 130 days. During the pre-test period, the feeding level of GAA gradually increased until the level set by the experiment was reached. Before the start of the experiment, the experimental steers were dewormed and ear tagged. They were fed once at 08:00 and 16:00 each day. Each group of steers had 12 automatic weighing feeders. The steers had free access to feed and water throughout the day.

Experiment 2. Fifteen 18-month-old Angus steers (575.60 ± 7.78 kg of BW) were selected and randomly assigned to three groups according to a one-factor randomized block design, with five steers per group. The group names, amount of additives, and the feeding method were the same as in Experiment 1. This experiment was conducted in Beijing in June, with a pre-test period of 7 days and a formal test period of 3 days (sampling period). During the pre-test period, the feeding level of guanidine acetic acid was gradually increased until the level set by the experiment was reached. During the pre-test period, the dry matter intake (DMI) of all steers was measured, and the appropriate feeding amount was determined according to the DMI for restricted feeding. All steers had free access to water.

### 2.3. Sample Collection and Measurements

Experiment 1. The steers were weighed before feeding on 0, 65, and 130 days of the test period, and the ADG of the test period was calculated. During the test period, the DMI of each steer was recorded by a random intake automatic recording system (Shanghai Zhenghong Animal Husbandry Machinery Equipment Co., Ltd., Shanghai, China). The FCE of each steer was calculated based on the ADG and DMI. A feed sample was taken every two weeks during the test period to determine the DM content, and a wind-dried sample was made for storage.

Rumen fluid from each steer via oral stomach tubing was collected before feeding on day 130. After filtering through four layers of gauze, it was dispensed into 15 mL centrifuge tubes for subsequent rumen fermentation parameter determination. The pH of the filtered rumen fluid was measured using a Testo 205 portable pH meter (Testo AG, Schwarzwald, Germany). The filtered rumen fluid was centrifuged at 8000× *g* for 15 min at 4 °C, and the supernatant was used to determine the concentrations of volatile fatty acids (VFAs) and ammonia nitrogen (NH_3_-N). The NH_3_-N concentration was determined by colorimetry [21], and the VFA concentration was determined by gas chromatography [22]. 

On day 130 before feeding, tail vein blood was collected from each steer and collected in 5 mL whole blood collection tubes and 5 mL anticoagulant blood collection tubes. White blood cell (WBC), red blood cell (RBC), hemoglobin (HGB), hematocrit (HCT), mean corpuscular volume (MCV), mean corpuscular hemoglobin (MCH), mean corpuscular hemoglobin concentration (MCHC), and platelet (PLT) levels of the cattle were determined by a hematology analyzer (Sysmex XN-1000). GAA, creatine, creatinine, and adenosine triphosphate (ATP) were determined using HPLC (Agilent HPLC 1200, Leadman Biochemistry Co., Ltd., Beijing, China) according to Shingfield and Offer [23]. Glucose (GLU), total protein (TP), albumin (ALB), globulin (GLB), alanine aminotransferase (ALT), aspartate aminotransferase (AST), alkaline phosphatase (ALP), total bilirubin (TBiL), direct bilirubin (DBiL), urine (UREA), uric acid (UA), blood ammonia (BA), and creatine kinase (CK) in the serum were measured using diagnostic kits (Leadman Biochemistry Co., Ltd., Beijing, China) and analyzed by an automated serum biochemical analyzer (Hitachi 7600). The activities of the oxidative and antioxidant were detected with kits (Nanjing Jiancheng Bioengineering Institute, Nanjing China) as previously described. In general, superoxide dismutase (SOD) activity was determined spectrophotometrically at 550 nm by the xanthine and xanthine oxidase system [24]. The malondialdehyde (MDA) measurement was based on the reaction with thiobarbituric acid in an acidic medium at 95 °C at 533 nm [25]. Glutathione peroxidase (GSH-Px) activity was determined at 550 nm by using 5,5′-dithiobis (2-nitrobenzoic acid) (DTNB) as a colorimetric probe [26]. Catalase (CAT) was detected with a spectrometer at a wavelength of 412 nm by reacting with a reduced form of glutathione [27]. The ferric reducing ability of a plasma assay measures the total antioxidant capacity (T-AOC) to reduce ferric ions to ferrous ions in the presence of a colorimetric probe [28]. The results are expressed as the change in absorbance at 593 nm.

Experiment 2. During the 3 days of the formal test period, TMR samples were collected before and after feeding every day, dried at 65 °C, and crushed for later testing. At the same time, fecal and urine samples of all steers were collected for 3 days during the formal test period.

The fecal collection process used the total fecal collection method. The experimenters watched the animals during the day and night for 3 days and collected the feces as they were excreted. The feces collected throughout the day were weighed and sampled according to 20% of the total weight, and 10% of the sample weight was added with tartaric acid to prevent nitrogen volatilization. The fecal samples collected every day were frozen at −20 °C, and after 3 days, all the fecal samples collected were mixed, weighed, dried at 65 °C, crushed, and stored at 4 °C for later testing.

Urine collection bags were used to collect urine from each steer throughout the day. The urine collection bag consisted of a urine collection funnel, a catheter, a urine collection bucket, and a latex tube. Before collecting urine, 200 mL of 6 N hydrochloric acid was added to the urine collection bucket. During the urine collection period, the urine collection bucket was shaken from time to time to ensure that the hydrochloric acid and urine were fully mixed to prevent nitrogen volatilization. The volume of urine collected every day was accurately measured with a graduated cylinder and then filtered through four layers of gauze and sampled at a ratio of 10%. After the end of the 3-day sampling period, the urine of each steer for 3 days was thoroughly mixed and stored at 4 °C for later testing. 

### 2.4. Chemical Analysis

The DM of TMR, feed residue, and fecal samples were determined after drying at 105 °C for 4 h (method 934.01; AOAC, 1997) [29], and the CP content was determined by the Dumas combustion method [30]. The neutral detergent fiber (NDF) was determined according to the method of Van Soest et al. [31]. The nitrogen content in urine was determined by the Kjeldahl method [32].

### 2.5. Calculation and Statistical Analysis

The apparent digestibility was calculated according to the following formula:

$$Da=\frac{Ia-Fa}{Ia}\times 100\%$$

where *Da* is the digestibility of nutrient *a*, *Ia* is the intake of nutrient a in the experimental diet, and *Fa* is the content of nutrient a in the feces.

Nitrogen retention rates are calculated based on the following formula:

$$N\;Retention=1-\frac{FecesN+UrineN}{N\;intake}\times 100\%$$

A one-way analysis of variance was performed using SPSS software (SPSS 26.0), and quadratic polynomial comparison and Duncan multiple comparisons were performed. *p* < 0.01 indicates extremely significant differences between groups, 0.01 < *p* < 0.05 indicates significant differences between groups, 0.05 < *p* < 0.1 indicates no significant differences but a trend of differences between groups, and *p* > 0.1 indicates no significant difference between groups nor any trend.

## 3. Results

### 3.1. Growth Performance and Rumen Fermentation

Table 2 illustrates the impact of varying doses of GAA on the growth performance of Angus steers. No significant differences were observed in DMI among the three treatment groups throughout the experiment, and differences among treatments for BW could not be detected. However, ADG exhibited a significant increase in the CGAA and HGAA groups compared to the CON group (*p* = 0.043). Furthermore, FCE was notably higher in the CGAA and HGAA groups compared to the CON group (*p* = 0.018).

Table 3 details the effects of different GAA doses on rumen pH, NH_3_-N, and volatile fatty acids (VFAs) in Angus steers. While no significant differences were observed in pH and total VFAs among the groups, a linear decrease in rumen NH_3_-N was evident with increasing GAA dosage (*p* = 0.062). The acetate concentration significantly decreased in the CGAA and HGAA groups compared to the CON group (*p* = 0.003), whereas propionate concentration significantly increased (*p* < 0.001). Additionally, the acetate:propionate ratio was significantly lower in the CGAA and HGAA groups compared to the CON group (*p* < 0.001).

### 3.2. Blood Indices

**Figure 1:**
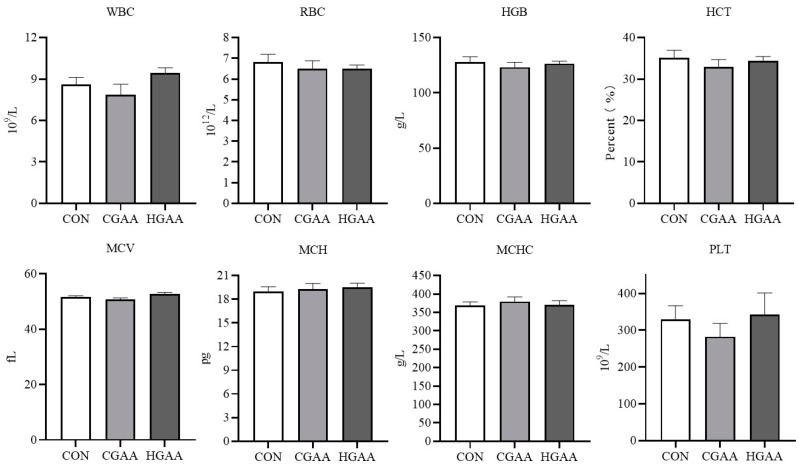
Effects of guanidinoacetic acid (GAA) supplementation on routine blood indices in Angus steers. WBC: white blood cell; RBC: red blood cell; HGB: hemoglobin; HCT: hematocrit; MCV: mean corpuscular volume; MCH: mean corpuscular hemoglobin; MCHC: mean corpuscular hemoglobin concentration; PLT: platelet. CON, control group; CGAA, conventional dose guanidinoacetic acid group; HGAA, high dose guanidinoacetic acid group.

**Figure 2:**
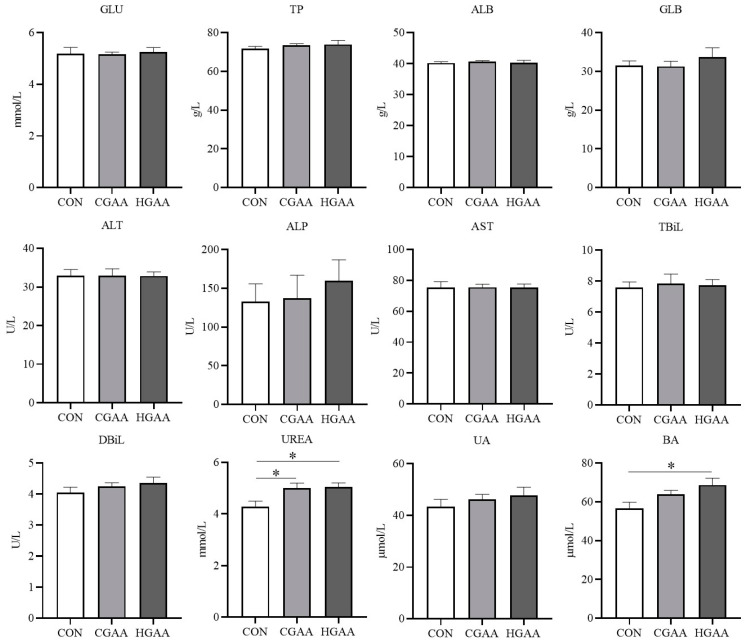
Effects of guanidinoacetic acid (GAA) supplementation on serum biochemical indicators in Angus steers. GLU = glucose; TP = total protein; ALB = albumin; GLB = globulin; ALT = alanine aminotransferase; AST = aspartate aminotransferase; ALP = alkaline phosphatase; TBiL = total bilirubin; DBiL = direct bilirubin; UREA = urine; UA = uric acid; BA = blood ammonia. CON, control group; CGAA, conventional dose guanidinoacetic acid group; HGAA, high dose guanidinoacetic acid group. * means 0.01 < *p* < 0.05.

**Figure 3:**
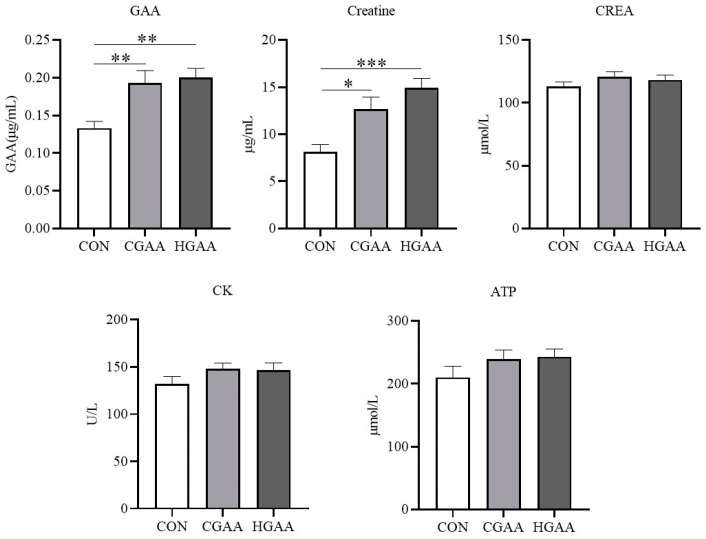
Effects of guanidinoacetic acid (GAA) supplementation on indicators of serum creatine metabolism in Angus steers. GAA = guanidinoacetic acid; CREA = creatinine; CK = creatine kinase; ATP = adenosine triphosphate. CON, control group; CGAA, conventional dose guanidinoacetic acid group; HGAA, high dose guanidinoacetic acid group. * means 0.01 < *p* < 0.05; ** means 0.001 < *p* < 0.01; *** means *p* < 0.001.

**Figure 4:**
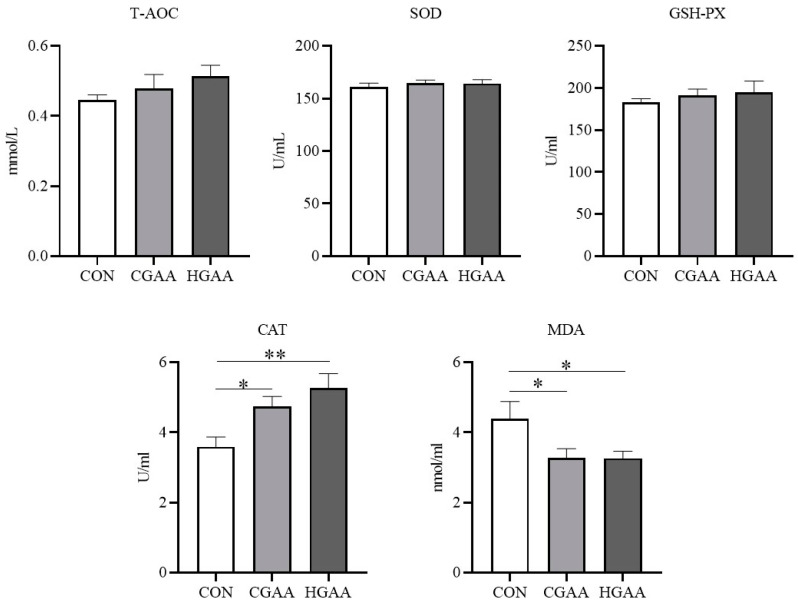
Effects of guanidinoacetic acid (GAA) supplementation on serum antioxidant indices in Angus steers. T-AOC = total antioxidant capacity; SOD = superoxide dismutase; GSH-Px = glutathione peroxidase; CAT = catalase; MDA = malondialdehyde. CON, control group; CGAA, conventional dose guanidinoacetic acid group; HGAA, high dose guanidinoacetic acid group. * means 0.01 < *p* < 0.05; ** means 0.001 < *p* < 0.01.

The addition of GAA did not significantly affect routine blood indices in Angus steers (Figure 1). However, Figure 2 demonstrates that the CGAA and HGAA groups exhibited significantly higher concentrations of UREA and BA compared to the CON group (*p* < 0.05). Results from serum creatine metabolism (Figure 3) revealed significantly higher concentrations of GAA and creatine in both the CGAA and HGAA groups compared to the CON group (*p* < 0.01). Figure 4 outlines the effect of GAA addition on serum antioxidant indexes in Angus steers. The CAT content was significantly higher in the CGAA and HGAA groups than in the CON group (*p* = 0.004), while MDA concentration was significantly lower in the CGAA and HGAA groups than in the CON group (*p* = 0.036).

### 3.3. Nutrient Digestion and Nitrogen Metabolism

Table 4 displays the effects of different GAA doses on nutrient digestibility and nitrogen metabolism in Angus steers. DM and CP digestibility were significantly higher in the CGAA and HGAA groups compared to the CON group (*p* < 0.05). There was also a tendency for NDF digestibility to be higher than in the CON group (*p* = 0.062). Despite no differences in DMI among the groups, feces N content in the CGAA and HGAA groups was significantly lower than that in the CON group (*p* = 0.034), resulting in a significantly higher nitrogen retention ratio (*p* = 0.024). Additionally, feces N/N excretion was significantly lower in the CGAA and HGAA groups compared to the CON group (*p* < 0.05), while urinary N/N excretion was significantly higher in the CGAA and HGAA groups compared to the CON group (*p* = 0.021).

## 4. Discussion

In animals, GAA is the only precursor substance for the synthesis of creatine; however, animals synthesize only 70 percent of their own creatine requirements and require exogenous supplementation [2,33]. Creatine is not present in plant-based feed ingredients, it is only in animal by-products [10], and since the feeding constraints of ruminants preclude the addition of any additives of animal origin, exogenous supplementation with GAA is an important modality to increase the creatine reserves in ruminants. Studies have shown that the regulatory effects of GAA on energy utilization and protein synthesis contribute to the growth performance of animals [11,13,15,34]. In the present study, it was found that the increase in ADG and FCE in the CGAA group compared to the control group indicated that GAA plays a key role in the growth of Angus steers, which is consistent with previous findings [14,19]. In addition, Liu et al. found that GAA improved milk production performance in Holstein cows [35], and these findings provide a database for the use of GAA in several cattle breeds. However, Angus steers with high doses of GAA added did not achieve higher growth performance, which may be related to methyl donor limitation. Creatine synthesis is a major consumer of methyl in the human body [6,36] due to the fact that guanidinoacetic acid is the recipient of a methyl group in order to form creatine [37]. Methyl donors required by beef cattle can be metabolically produced through dietary catabolism or exogenously added [38], and methyl donors from catabolism of the diets in this study may not be sufficient to meet the requirements of beef cattle in the HGAA group, which may have resulted in the failure to achieve a linear stacking effect in growth performance with the addition of high doses of GAA. In conclusion, the results of GAA in improving growth performance and feed efficiency of Angus steers without increasing feed intake side by side reflect its excellent economic value as a growth-promoting additive.

In terms of rumen fermentation parameters, rumen pH was in the normal range and did not differ between groups after feeding GAA, suggesting that the addition of GAA does not negatively affect rumen health [39]. The results of no difference in TVFA concentration among the three groups also echoed the results of pH. The concentration of NH_3_-N in the present study tended to decrease linearly with increasing GAA concentration. Li et al. showed the same results as the present study; they found that the addition of GAA significantly reduced the concentration of NH_3_-N in the rumen of Angus bulls, increased the abundance of total bacteria and total fungi, and decreased the abundance of total protozoa in the rumen [14]. However, the opposite results were found by Liu et al., who found a significant increase in ruminal NH_3_-N in Angus bulls following the addition of GAA [19]. The different trends in rumen NH_3_-N may be related to multiple factors such as rumen microbes, feed composition, and growth stage of the cattle. It has been shown that the fate of NH_3_-N in the rumen is mainly microbial protein synthesis and the urea N cycle [40], and the decrease in rumen NH_3_-N concentration after the addition of GAA in this study may be related to the increased utilization of NH_3_-N by rumen microorganisms. Meanwhile, the decrease in the concentration of acetic acid and the increase in the concentration of propionic acid suggest that the addition of GAA may have altered the fermentation in the rumen, which can promote more efficient utilization of nutrients by gut microorganisms [14]. This provides a basis for subsequent exploration of nutrient digestibility.

In this study, the addition of GAA did not affect routine blood markers in Angus steers, which is consistent with the results of a previous study [41]. This suggests that GAA does not have a negative effect on the health of Angus steers. BA is mainly derived from ammonia produced by the deamidation of dietary proteins and amino acids, which are transported to the liver to be synthesized into urea via the urea cycle [42]. The addition of GAA increased the blood concentrations of UREA and BA, but this increase was within a reasonable range [43]. However, changes in BA and UREA concentrations moved in the opposite direction to changes in ruminal NH_3_-N concentrations. There are two possible reasons for this: one is that the utilization of NH_3_-N by the microbial community in the rumen may be enhanced as we mentioned before, and the other is that since GAA improves the digestibility of CP, it may lead to the production of more ammonia into the blood, making the ammonia concentration in the blood higher. Overall, the changes in blood BA and UREA concentrations suggest that the addition of GAA may have an effect on nutrient digestion and absorption and nitrogen metabolism in steers. In this study, we found that the contents of GAA and creatine in both CGAA and HGAA groups were higher than those in the CON group, which indicated that GAA addition was not completely degraded by microorganisms in the rumen but was able to enter into the bloodstream and could increase the creatine content in the organisms of Angus steers, which could also explain the increase in the growth performance of Angus steers after the addition of GAA. However, the HGAA group did not have significantly higher levels of GAA and creatine than the CGAA group, which is consistent with the trend of the growth performance results, which may be due to methyl donor limitation. The more obvious conclusion is that Angus steers do not need a higher dose of GAA than the conventional dose. In our study, the addition of GAA increased CAT levels and decreased MDA levels in the blood of Angus steers, suggesting that GAA enhanced the antioxidant capacity of steers. This is consistent with previous studies [18]. Creatine has been reported to have the ability to remove O2- [44], and the changes in creatine concentration in this study coincided with the changes in antioxidant indices. This suggests that GAA may also act as a growth promoter by enhancing the antioxidant capacity of the beef cattle organism.

Based on the changes associated with rumen fermentation and blood indices in this study, it is necessary to investigate the effects of GAA on nutrient digestibility in Angus steers. In the present study, the digestibility of DM and CP increased with the addition of GAA, and this increase suggests that supplementation with GAA positively affects the assimilation and utilization of dietary nutrients and may contribute to the improvement of the overall nutrient supply to the animals. In addition, a significant increasing trend in NDF digestibility was also observed, and although this trend did not reach statistical significance, it hints at a potential positive effect of GAA supplementation on the utilization of fiber components of the diet, which deserves to be further explored in future studies. It is worth noting that previous studies are consistent with our results, although not in terms of digestibility as measured by the full-collection fecal method. For example, previous studies have confirmed that the addition of GAA increases the apparent digestibility of dry matter, crude protein, NDF, and ADF in Angus bulls [14,19], while a study has confirmed that the addition of GAA increases the apparent digestibility of dry matter, crude protein, NDF, and ADF in Holstein dairy cows [35]. In addition, studies have shown that GAA can increase nutrient digestibility in pigs [11] and broilers [13]. This demonstrates the broad species applicability of GAA as a growth-promoting additive.

In conjunction with changes in NH_3_-N and blood ammonia, in order to further investigate the effects of GAA on nitrogen metabolism, a digestive metabolism experiment was used in this study with the aim of focusing on the effects of GAA on nitrogen metabolism in Angus steers. This is because previous studies have found that the addition of GAA does affect N metabolism in the ruminant organism [17]. In this study, it was found that the addition of GAA decreased the N content in the feces, while N retention was significantly increased. This suggests that GAA supplementation improves dietary N retention and hence N utilization efficiency in Angus steers, which was confirmed by the results of feces N/N excretion in the CGAA and HGAA groups. In this study, there was no difference in urinary N between the three groups, but urinary N/N excretion was significantly higher in the CGAA and HGAA groups, which echoed the reduction in fecal nitrogen. Animal feces and urinary nitrogen are potential sources of environmental pollution [45], and the results of changes in urinary and feces nitrogen suggest that the addition of GAA during feeding beef cattle seems to appear to be a good thing for the environment. The use of GAA may improve the efficiency of protein utilization by steers, reducing the emission of unutilized nitrogen and contributing to the efficiency of nitrogen use in the ecosystem. In addition, GAA makes the resource more sustainable in its use. Reducing feed waste and nitrogen emissions helps reduce the environmental burden of production. It is important to note that the specific environmental impacts of GAA depend on a number of factors, including feeding management, feed composition, and animal physiological status. For the assessment of environmental sustainability, it is recommended to follow up with detailed research and monitoring in studies to fully understand the environmental impacts of GAA supplementation in beef cattle farming.

## 5. Conclusions

The present study showed that both 0.8 g/kg and 1.6 g/kg of GAA promoted creatine synthesis and improved nutrient digestibility and the nitrogen retention ratio, which, in turn, improved growth performance in Angus steers and also showed some antioxidant potential. However, the addition of higher doses of GAA did not show better results, which may be related to methyl donor limitation. Overall, these findings underscore the considerable potential of GAA in enhancing ruminant productivity. Future research should prioritize exploring the potential effects of GAA in combination with methyl donors on beef cattle performance and meat quality.

## Figures and Tables

**Table 1:** The composition of dietary raw materials and nutrients (DM basis, %).

Ingredient Composition	Content	Chemical Composition ^2^	Content
Whole corn silage	45.00	DM	93.46
Corn stalks	15.00	CP	12.61
Corn	25.15	NDF	32.74
Soybean meal	3.15	Ca	0.67
Cottonseed meal	6.75	P	0.40
CaHPO_4_	0.90	ME/(Mcal/kg)	2.52
NaHCO_3_	1.35	NE_m_/(Mcal/kg)	1.64
NaCl	0.90	NE_g_/(Mcal/kg)	1.05
Premix ^1^	1.80		
Total	100.00		

^1^ The premix provided the following per kg of diets: Cu 10 mg, Fe 65 mg, Mn 30 mg, Zn 25 mg, I 0.5 mg, Se 0.1 mg, Co 0.1 mg, V_A_ 4000 IU, V_D_ 500 IU, V_E_ 40 IU. ^2^ DM, dry matter; CP, crude protein; NDF, neutral detergent fiber; ME, NE_m_, and NE_g_ were calculated values, while the others were measured values.

**Table 2:** Effects of guanidinoacetic acid (GAA) supplementation on production performance in Angus steers.

Items ^1^	Supplementary Groups ^2^			SEM	*p*-Value ^3^
	CON	CGAA	HGAA		
DMI (kg/day)	9.66	9.98	10.13	0.17	0.508
BW (kg)					
0 d	482.25	488.08	486.58	6.57	0.935
65 d	545.75	561.67	557.58	7.32	0.667
130 d	599.67	624.75	622.92	7.96	0.368
ADG/(kg/d)	0.90 ^b^	1.05 ^a^	1.05 ^a^	0.03	0.043
FCE (kg/kg)	0.09 ^b^	0.11 ^a^	0.10 ^a^	0.002	0.018

^1^ DMI, dry matter intake; BW, body weight; ADG, average daily gain; FCE, feed conversion efficiency, FCE= ADG/DMI. ^2^ CON, control group; CGAA, conventional dose guanidinoacetic acid group; HGAA, high dose guanidinoacetic acid group. ^3^ *p* < 0.05 means highly significant difference; 0.05 < *p* < 0.10 means significant difference; *p* > 0.10 means no significant difference. ^a,b^ Indicate significant differences (*p* < 0.05).

**Table 3:** Effects of guanidinoacetic acid (GAA) supplementation on rumen fermentation parameters in Angus steers.

Items ^1^	Supplementary Groups ^2^			SEM	*p*-Value ^3^
	CON	CGAA	HGAA		
pH	6.85	6.78	6.77	0.02	0.111
NH_3_-N, mg/100 mL	5.09	4.77	4.38	0.13	0.062
TVFA, mmo/L	63.49	65.99	65.10	0.49	0.120
VFA, %					
Acetate	70.30 ^a^	67.98 ^b^	68.61 ^b^	0.30	0.003
Propionate	16.23 ^b^	18.66 ^a^	18.16 ^a^	0.28	<0.001
Isobutyrate	0.72	0.65	0.67	0.03	0.659
Butyrate	10.44	10.30	10.30	0.12	0.894
Isovalerate	1.60	1.67	1.50	0.04	0.189
Valerate	0.72	0.74	0.75	0.02	0.901
Acetate:Propionate	4.35 ^a^	3.65 ^b^	3.79 ^b^	0.08	<0.001

^1^ TVFA, total volatile fatty acid. ^2^ CON, control group; CGAA, conventional dose guanidinoacetic acid group; HGAA, high dose guanidinoacetic acid group. ^3^ *p* < 0.05 means highly significant difference; 0.05 < *p* < 0.10 means significant difference; *p* > 0.10 means no significant difference. ^a,b^ Indicate significant differences (*p* < 0.05).

**Table 4:** Effects of guanidinoacetic acid (GAA) supplementation on nutrient digestibility and nitrogen metabolism in Angus steers.

Items ^1^	Supplementary Groups ^2^			SEM	*p*-Value ^3^
	CON	CGAA	HGAA		
Digestibility, %					
DM	69.72 ^b^	72.41 ^a^	71.81 ^a^	0.005	0.023
NDF	50.91	54.53	55.78	0.009	0.062
CP	69.71 ^b^	73.31 ^a^	72.76 ^a^	0.005	0.002
Nitrogen metabolism					
DMI, kg/d	6.73	6.67	6.70	0.087	0.966
N intake, g/d	129.59	128.43	128.86	1.669	0.966
Feces N, g/d	39.27 ^a^	34.29 ^b^	35.09 ^b^	0.889	0.034
Urine N, g/d	54.23	55.90	55.73	0.888	0.730
N retention, g/d	36.08	38.24	38.03	0.632	0.328
N retention ratio, %	27.85 ^b^	29.77 ^a^	29.52 ^a^	0.003	0.024
N excretion, g/d	93.51	90.19	90.83	1.286	0.571
Feces N/N excretion, %	42.00 ^a^	38.02 ^b^	38.65 ^b^	0.007	0.021
Urinary N/N excretion, %	58.00 ^b^	61.98 ^a^	61.35 ^a^	0.007	0.021
Urine N/N intake, %	41.86	43.54	43.24	0.005	0.331

^1^ DM = dry matter; NDF = neutral detergent fiber; CP = crude protein; DMI = dry matter intake. ^2^ CON, control group; CGAA, conventional dose guanidinoacetic acid group; HGAA, high dose guanidinoacetic acid group. ^3^ *p* < 0.05 means highly significant difference; 0.05 < *p* < 0.10 means significant difference; *p* > 0.10 means no significant difference. ^a,b^ Indicate significant differences (*p* < 0.05).

## Data Availability

All the data are presented in the text and tables of this manuscript.
